# Beyond Disability: Older Adult Perceptions of Successful Aging

**DOI:** 10.1093/geroni/igaf122.699

**Published:** 2025-12-31

**Authors:** Payton Rule, Jennifer Pearlstein, Emily Willroth

**Affiliations:** Washington University in St. Louis, St. Louis, Missouri, United States; WashU Medicine, St. Louis, Missouri, United States; Washington University in St. Louis, St. Louis, Missouri, United States

## Abstract

High physical and mental function and the absence of disability have long been included as key components of most successful aging models. However, prior work suggests these components may not be necessary for successful aging. Moreover, this definition focuses on individual abilities without acknowledging the role of inclusive environments (e.g., no stairs, large print materials) in promoting participation and successful aging. To address these limitations, we created a broader and more inclusive definition of successful aging as continued engagement in the activities one needs and wants to participate in—with or without accommodation, assistance, and environmental changes. To empirically evaluate this proposed definition, we recruited 427 older adults, with and without disabilities, to complete an online survey. Participants rated how closely their perception of successful aging aligned with three existing definitions and our proposed definition. They also answered questions regarding their physical health, wellbeing, and perceptions of their own aging. A series of linear regression analyses and paired-samples t-tests were conducted to assess older adults’ perceptions of our proposed definition compared to existing definitions of successful aging. Additional analyses examined associations between aging successfully according to each definition and subjective wellbeing and subjective successful aging. Results indicated that older adults’ perceptions of successful aging aligned significantly better with our proposed definition than with existing physiologically-focused definitions. Furthermore, our proposed definition was significantly associated with wellbeing and individuals’ subjective sense of aging successfully. This talk will discuss implications of this disability-inclusive successful aging conceptualization for future research, clinical practice, and policy.

